# Genetic variation and phylogenetic analysis of Indonesian indigenous catfish based on mitochondrial cytochrome oxidase subunit III gene

**DOI:** 10.14202/vetworld.2019.896-900

**Published:** 2019-06-27

**Authors:** Rini Widayanti, Aris Haryanto, Wayan Tunas Artama, Suhendra Pakpahan

**Affiliations:** 1 Department of Biochemistry and Molecular Biology, Faculty of Veterinary Medicine, Gadjah Mada University, Yogyakarta, Indonesia; 2 Biology Study Program, Faculty of Biotechnology, Duta Wacana Christian University, Yogyakarta, Indonesia

**Keywords:** cytochrome c oxidase-III, *Hemibagrus*, Indonesian indigenous catfish, mitochondrial DNA, phylogenetic

## Abstract

**Aim:**

This study aimed to analyze the genetic variation and phylogenetic reconstruction of Indonesian indigenous catfish using mitochondrial cytochrome oxidase subunit III sequences.

**Materials and Methods:**

A total of 19 samples of catfish were collected from seven rivers (Elo [EM], Progo [PM], Kampar [KR], Musi [MP], Mahakam [MS], Kapuas [KS], and Bengawan Solo [BSBJ]) in five different geographical locations in Indonesia. The genome was isolated from the tissue. Mitochondrial DNA cytochrome oxidase subunit III was amplified using polymerase chain reaction (PCR) with CO3F and CO3R primers. The PCR products were sequenced and continued to analyze genetic variation and phylogenetic relationship using MEGA version 7.0 software.

**Results:**

Cytochrome c oxidase (*COX*)-III gene sequencing obtained 784 nucleotides encoding 261 amino acids. Sequenced *COX-III* gene fragments were aligned along with other catfish from Genbank using ClustalW program and genetic diversity among species was analyzed using the MEGA Version 7.0 software. Among all samples, there were substitution mutations at 78 nucleotide sites, and there were 14 variations in amino acids. Catfish from PM, KR, MP, and KS had the same amino acids as *Hemibagrus nemurus* (KJ573466.1), while EM catfish had eight different amino acids and catfishBSBJhad 12 different amino acids.

**Conclusion:**

Indonesian catfish divided into four clades. BBSJ Catfish were grouped with *Pangasianodon gigas*, EM catfish were grouped with *Mystus rhegma*, and KS catfish were grouped with *Hemibagrus spilopterus*, while catfish MS, KR, PM, andMP were grouped with *H. nemurus*.

## Introduction

Indonesia has a very high diversity of fish species. Baung fish are one of the Indonesian indigenous catfish and belong to *Hemibagrus*. Baung fish are found in rivers on Java, Sumatra, and Kalimantan islands. At present, Baung fish have begun to become endangered in the wild. Species of catfish are widely distributed throughout India, Southern China, and Southeast Asia [1]. Species of the catfish genus *Hemibagrus* are large and spread in all rivers of the Indonesian archipelago [2]. At present, several species of catfish have been cultured for food in Indonesia, namely *Hemibagrus nemurus, Hemibagrus hoevenii*, and *Hemibagrus fortis*, but other species still live wild in nature. Baung fish are one of the freshwater aquaculture commodities in Indonesia that have important economic value [3]. These fish have gained popularity among Indonesians due to their good flavor and high nutritional value [4], and it is commercially cultured in Asia [5].

*Hemibagrus* is related to *Clarias* spp.; therefore, the morphology of *Hemibagrus* is very similar to *Clarias* spp. The population of Baung fish is now increasingly scarce because it is constantly being caught from nature without any conservation by the community. Pollution in river water also decreases Baung fish populations. However, research on Baung fish is still lacking. Their existence is increasingly threatened by extinction because Baung fish are still very difficult to cultivate. Therefore research into the genetics and ecology of Baung fish is essential to prevent potential extinction and develop a successful conservation strategy.

Mitochondrial DNA (mtDNA) sequences are widely used in molecular evolutionary and phylogenetic relationship studies within and between vertebrate species [6]. Cytochrome c oxidase (*COX*) is the terminal enzyme of the mitochondrial respiratory chain and contains 14 protein subunits in mammals. *COX* has three subunits which are encoded by mtDNA (*COX* subunit I, subunit II, and subunit III) [7]. The *COX* subunit III protein is an important element for regulating the efficiency of proton translocation in cytochrome oxidase over several turnovers [8]. *COX* subunit III has been used in several genetic diversity studies including human [9], chicken [10], brown seaweed [11], and *Babesia* [12]. The function of *COX-III* gene is very important for the respiratory chain and is highly conserved among species [10].

The objective of this study was to analyze *COX* subunit III single-nucleotide polymorphisms of Baung fish and their phylogenetic relationship with populations from different parts of Indonesia using *COX* subunit III as the mitochondrial marker with available GenBank records. Furthermore, genetic variability of catfish of this region of Indonesia was inferred from mtDNA sequences to get the DNA barcoding of catfish among different populations.

## Materials and Methods

### Ethical approval

This study was approved by the Animal Ethics Committee for using Animal and Scientific Procedures in Faculty of Veterinary Medicine, Gadjah Mada University, Indonesia.

### Catfish collections

**Figure-1:**
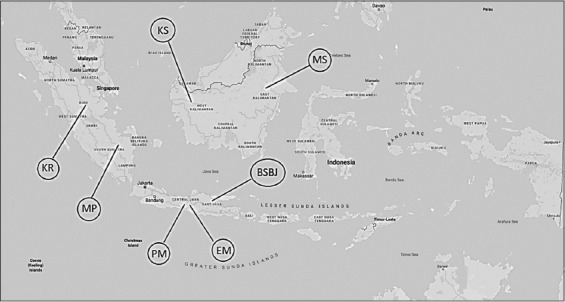
Location map of catfish collection (Source: Google maps).

Baung fish (catfish) specimens were collected from seven different rivers (Elo [EM], Progo [PM], Kampar [KR], Musi [MP], Mahakam [MS], Kapuas [KS], and Bengawan Solo [BSBJ]) from five provinces on three islands (Sumatra, Java, and Kalimantan) (Figure-1). The number of each kind is shown in Table-1. The catfish samples were unrelated genetically because they were taken individually from nature.

**Table-1:** Origin and number of samples used in this study.

River	Province	Number of samples	Sample codes
Elo	Magelang, Central Java	2	EM1, EM2
Progo	Magelang, Central Java	3	PM1, PM2, PM3
Kampar	Pekanbaru, Riau, Sumatra	3	KR1, KR2, KR3
Musi	Palembang, South Sumatra	3	MP1, MP2, MP3
Mahakam	Samarinda, East Kalimantan	3	MS1, MS2, MS3
Kapuas	Sintang, West Kalimantan	2	KS1, KS2
Bengawan Solo	Bojonegoro, East Java	3	BSBJ1, BSBJ2, BSBJ3

All individuals were identified based on morphological characteristics and sample tissues preserved in RNA latter buffer (Qiagen) were used for total DNA extraction.

### DNA extraction and amplification

Total DNA was extracted with gSYNC™ DNA Mini Extraction Kit (Geneaid Biotech Ltd., Taiwan) according to the manufacturer’s instructions and total DNA stored at −20°C until use. DNA fragments of the target region were amplified by polymerase chain reaction (PCR) using a pair primer, BaungCO3F 5’ taccatatacatttacaccaa3’ and BaungCO3R 5’ctttccttggattttaacca3’.

The primer was designed using Primer3 output program (http://www-genome.wi.mit.edu/cgi.bin/primr3.cgi/results_from-primer3) based on mitochondrial genetic sequence data of *H. nemurus* (access number KJ573466.1) and *Mystus vittatus* (KX177968.1).

The total volume of reaction was 50 µL 2 µl DNA template, 21 µL distilled water, 1 µL (10 pmol) of each primer, and 25 µL master mix (Kapa2G ready mix, 1^st^ Base). Reaction cycles consisted of an initial denaturing step at 94°C for 5 min, followed by 35 cycles at 94°C for 30 s, 46°C for 45 s, and 72°C for 90 s, with a final extension at 72°C for 5 min using an Infinigen thermocycler. DNA amplifications were analyzed by 1% agarose gel electrophoresis for genotyping.

### Sequences and phylogenetic analysis

The purified PCR products were sequenced directly by 1^st^ Base Sequencing INT (Singapore). The DNA forward and reverse sequences of *COX-III* gene were aligned using ClustalW, edited and then multiple alignments were performed with data linked to *H. nemurus* and other catfish from other countries deposited in GenBank. The fragments of *COX-III* were analyzed on 784 nucleotides. The genetic distance was conducted using Kimura 2-parameter method and phylogenic analyses based on *COX-III* sequence data were conducted by neighbor-joining (NJ) using MEGA program version 7.0. [10]. Bootstrap method for genetic distance analysis was conducted with 1000 replicates. A phylogenetic tree was constructed based on *COX-III* sequences and catfish sequences from other countries were used in order to reveal relationships and clusters between catfish.

## Results

### Fragment sequence length and base constitution of mtDNA COX-III in Indonesian catfish

**Figure-2:**
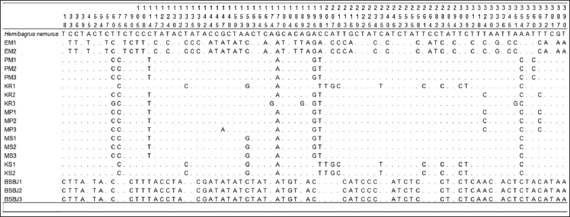
Polymorphic sites of Indonesian catfish from site 1 to 390. Identity with the first sequences is denoted by a dot.

**Figure-3:**
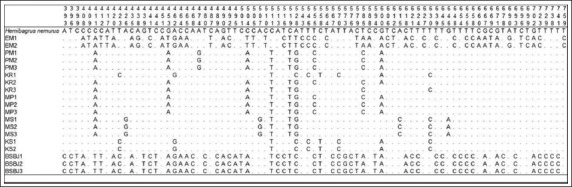
Polymorphic sites of Indonesian catfish from site 391 to 784. Identity with the first sequences is denoted by a dot.

Partial *COX-III* sequences with 784 bp were determined for each of the 19 samples of Indonesian catfish. Among the total samples, there were 78 sites of substituted nucleotides and there was no deletion or insertion of nucleotides. Variation occurred due to the substitution between nucleotides (Figures-2 and 3). The average percentage of nucleotide T, C, A, and G from each sample location was as follows: EM (28.7%, 27.6%, 27.8%, 15.9%); PM (29.8%, 27.2%, 26.3%, 16.7%); KR (29.7%, 27.3%, 26.4%, 16.6%); MP (29.7%, 27.3%, 26.5%, 16.5%); MS (30.1%, 27.2%, 25.6%, 17.1%); KS (29.5%, 27.9%, 25.8%, 16.8%) and BSBJ (27.4%, 29.2%, 27.7%, 15.7%).

### Variation in amino acids sequences

Fragment *COX-III* sequences of Indonesian catfish were translated to amino acid. A total of 261 amino acids for each individual were analyzed with reference to *H. nemurus* (KM454860.1). There were 14 amino acid variations in all Indonesian catfish. Catfish from PM, KR, MP, and KS had identical amino acids with *H. nemurus* (KJ573466.1), while EM had eight different amino acids and BSBJ had 12 different amino acids (Table-2).

**Table-2:** Amino acid variation in *COX-III* gene of Indonesian catfish.

Catfish	Amino acid sequence													

					1	1	1	1	1	2	2	2	2	2

	4	4	5	6	4	5	5	6	7	1	1	2	2	2

	4	8	5	2	7	1	5	4	1	6	9	0	3	4
*Hemibagrus nemurus*	T	I	L	I	A	L	A	A	L	A	F	F	V	R
EM1	.	M	.	.	.	I	E	T	.	.	L	L	I	Q
EM2	.	M	.	.	.	I	E	T	.	.	L	L	I	Q
PM1	.	.	.	.	.	.	.	.	.	.	.	.	.	.
PM2	.	.	.	.	.	.	.	.	.	.	.	.	.	.
PM3	.	.	.	.	.	.	.	.	.	.	.	.	.	.
KR1	.	.	.	.	.	.	.	.	.	.	.	.	.	.
KR2	.	.	.	.	.	.	.	.	.	.	.	.	.	.
KR3	.	.	.	.	.	.	.	.	.	.	.	.	.	.
MP1	.	.	.	.	.	.	.	.	.	.	.	.	.	.
MP2	.	.	.	.	.	.	.	.	.	.	.	.	.	.
MP3	.	.	.	.	.	.	.	.	.	.	.	.	.	.
MS1	.	.	.	.	.	.	.	.	.	T	.	.	.	.
MS2	.	.	.	.	.	.	.	.	.	T	.	.	.	.
MS3	.	.	.	.	.	.	.	.	.	T	.	.	.	.
KS1	.	.	.	.	.	.	.	.	.	.	.	.	.	.
KS2	.	.	.	.	.	.	.	.	.	.	.	.	.	.
BSBJ1	S	V	Y	V	S	.	E	T	F	.	L	L	I	Q
BSBJ2	S	V	Y	V	S	.	E	T	F	.	L	L	I	Q
BSBJ3	S	V	Y	V	S	.	E	T	F	.	L	L	I	Q

*COX*: Cytochrome c oxidase

### Phylogenetic relationship in Indonesian catfish and some catfish species

**Figure-4:**
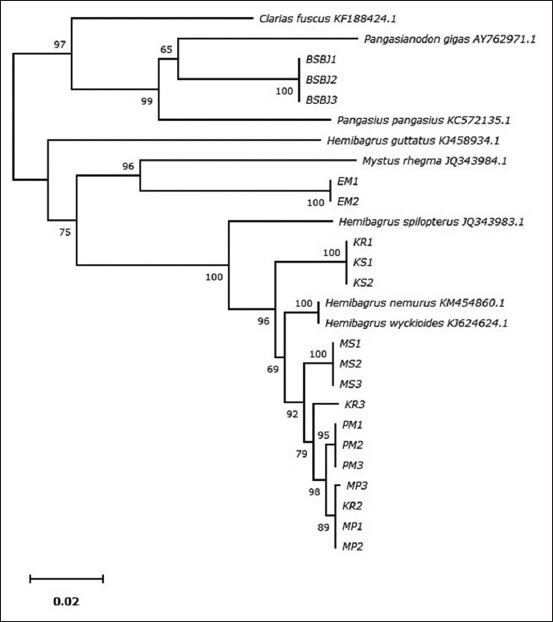
Phylogenetic tree of Indonesian catfish was constructed by the neighbor-joining method.

The first analysis aligned all sequences of each sample by the ClustalW program and continue with translating to amino acids by the BLAST program on NCBI (GenBank). The second analysis constructed the phylogenetic tree used the NJ algorithm in MEGA 7.0 [13] to develop a taxon identification phenogram of Indonesian catfish. A phylogenetic tree of 19 individual Indonesian catfish and some catfish from other countries (GenBank) was constructed (Figure-4). Variation of nucleotides or amino acids occurred due to the substitution among nucleotides [6]. The phylogenetic tree revealed that Indonesian catfish divided into four clades together with the catfish from other countries. BBSJ catfish were grouped with *Pangasianodon gigas*, EM catfish were grouped with *Mystus rhegma*, and KS catfish were grouped with *Hemibagrus spilopterus*, while MS, KR, PM, and MP catfish were grouped with *H. nemurus*.

## Discussion

### Divergence of morphological and genetic

Indonesia is a tropical country that has many rivers that have many types of catfish. The objective of this study was to identify the taxonomy and molecular characterization of Indonesian catfish. Most Indonesian people called these fish with the same name, Baung fish. Determining species of catfish is quite difficult based on morphological characteristics because catfish consists of many closely related types. Morphological similarity was largely confirmed by the analysis of the mitochondrial *COX-III* sequences. Combining morphological and genetic analyses to elucidate taxonomy will better elucidate the diversification and evolutionary relationships within this specious group of fishes [14,15]. The catfish commonly found throughout fresh- and brackish-water bodies in Asian and African continents, include more than 200 species in 17 genera and are one of the largest catfish families presently recognized [16]. *H. nemurus* is native to Asian waters and widely distributed throughout Malaysia, Indonesia, Cambodia, Laos, Thailand, and Vietnam [17]. The morphological characters are commonly used for identification of species in other catfish groups, but the similarity and wide distribution will complicate for identification of catfish. Therefore, the molecular identification and phylogenetic reconstruction of *Hemibagrus* are needed [18]. In this study, EM and BSBJ catfish obtained from Java Island had some different amino acids compared to catfish from other islands. These results provide evidence that EM and BSBJ were different species with *H. nemurus*. Amino acids of EM catfish showed some divergence from other catfish; there were only six amino acids common to all 14 variable sites. BSBJ catfish had 12 amino acids different to *H. nemurus* and MS catfish had only one different amino acid.

### Phylogeographic information of Indonesian catfish

The samples of this study were obtained from local people in different rivers and provinces. Baung is a term given by the local community for one type of catfish mentioned above. Based on previous studies about catfish molecular phylogenetics and phylogeo graphics, *H. nemurus* in Southeast Asia has extensive genetic subdivision of the group [19-22]. In the present study, in order to construct the phylogenetic tree, genetic data of some species of *Siluriformes* were obtained from GenBank and compared with Indonesian catfish. It was found that MS, KR, PM, and MP catfish formed one clade with *H. nemurus* and *H. wyckioides* species (bootstrap support 69% NJ). However, one sample (KR1) of KR was split into the KS group. Catfish KR1 and KS were formed into one clade and had genetic similarities with *H. splopterus*. EM had genetic similarities with *M. rhegma*, whereas BSBJ had genetic similarities with *P. gigas*. These results revealed that all samples in this study divided into four species of catfish. PM and MP are the last Indonesian catfish to form the most recent common ancestor in the *Hemibagrus* group. Dodson and Lecomte [22] have been investigated Indonesian catfish based on mtDNA cytochrome b (Sumatra, Java, and Borneo) and reported that Indonesian catfish consisted of *Hemibagrus planiceps* from Java*, Hemibagrus bongan* (Borneo), and *Hemibagrus velox* (Sumatra) species. Their component species were also generally congruent in morphological and genetic analyses. The geological events influencing the Indochinese mainland and the Sunda Islands have permitted both speciation and differential dispersal depending on the biology of the species. Hybridization alters mtDNA and creating recent common ancestors, so it will be difficult to clearly distinguish groups morphologically. Therefore, phylogenetic analysis is needed based on nuclear DNA.

## Conclusion

Based on *COX*-III genes, Indonesian catfish divided into four clades. BBSJ Catfish were grouped with *P. gigas*, EM catfish were grouped with *M. rhegma*, and KS catfish were grouped with *H. splopterus*, while catfish MS, KR, PM, and MP were grouped with *H. nemurus*.

## Authors’ Contributions

RW designed and collected samples of Baung fish for this study. SP carried out the study in the laboratory and wrote the manuscript. AH and WTA revised the manuscript and advised on this study. All authors have read and approved the final manuscript.
